# On the Use of Anæsthetics in Midwifery

**Published:** 1849-04

**Authors:** J. Y. Simpson

**Affiliations:** Edinburgh


### MEDICAL EXAMINER,

AND

### RECORD OF MEDICAL SCIENCE.

NEW SERIES.—NO. LII.—APRIL, 1849.

### ORIGINAL COMMUNICATIONS.

*On the use of Anæsthetics in Midwifery.* By J. Y. Simpson, M. D., of Edinburgh.

Editors of the Examiner:

Gentlemen,—I received by the last steamer, a letter from Professor Simpson, accompanying the observations on anæsthetics, which I herewith transmit.

As you were good enough to publish Dr. Simpson's former letter and my reply, I hope you will give publicity to this, his rejoinder; particularly as I have his permission to send it to the press.

I shall not occupy your pages with any remarks on the Professor's strictures. All my respect for his genius, and industry, and truthfulness, has, as yet, been insufficient to induce me to give chloroform in my obstetric practice. I have not had a case in which it appeared to me to be required by the state of the patient.

It is probable that, if I should be compelled to perform the operation of Turning in some shoulder presentation, with total loss of the liquor amnii, and a powerfully contracting uterus, I may make use of the anæsthetic. Should this happen, I shall make haste to report the result to the Edinburgh professor.

Meanwhile I gladly submit Dr. S.'s remarks to the readers of your journal, and remain very respectfully, &c.,

Charles D. Meigs.

*Philadelphia,* March 20th, 1849.

*Starbank, by Edinburgh,* 1st August, 1848.

My Dear Sir,—A few days ago, I saw your excellent epistle to me on the use of anæsthesia in midwifery, extracted in an abridged form from the Philadelphia Medical Examiner into the London Medical Gazette and Lancet. It reminded me, that amid other avocations and work, I had hitherto indolently omitted to answer the objections in your able and kind letter. And I feel that I am the more to blame for this neglect on one account, viz.: that as in your own country, so also in ours, there are few or no living obstetricians whose opinions and name carry, and deservedly carry, more weight with them than yours. Be so good, then, as to bear with me now for a few minutes, while I endeavour to state in what respects I am inclined to demur to your arguments against anæsthetic midwifery.

On reperusing (as I have just done) your esteemed letter, it appears to me that in it you ground your opposition to the adoption of anæsthesia in midwifery, upon four or five different arguments, although you do not specialize them. I shall notice each of these arguments separately. You have not placed them in any particular order. I shall begin first with the one which you placed last.

1. *You object to anæsthesia in deliveries requiring "chirurgical intervention; and especially in forceps operations, on the ground that the sensations of the patient afford us our best guide for the introduction of the instrument.*

In order to introduce the forceps with the greatest safety to the mother, you state, that (to quote your own words,) "the best guide of the accoucheur is the reply of the patient to his interrogatory, 'Does it hurt you?' The patient's reply 'Yes, or No,' are, (you observe,) worth a thousand dogmas and precepts. I cannot, therefore, (you continue,) deem myself justified in casting away my safest and most trustworthy diagnosis for the questionable equivalent of ten minutes exemption from pain, which, even in this case, is a physiological pain.

In answer to this novel objection, you will excuse me when I say it, (for I say it most conscientiously,) that I think every man who ventures to use the forceps in any midwifery case, ought to know the anatomy of the parts implicated, a thousand fold better than you here presuppose. You would have the accoucheur guide his instrument, not so much by his own anatomical knowledge, as by the feelings and sensations of his patient. In this, as in other points, relative to any novel question in practice, we can often, it appears to me, best perceive the soundness or unsoundness of our views upon it by considering and contrasting them with our established views on other analogous questions, regarding which, the opinions of the profession have been long fixed and determined. Now, what would the surgical world, at this time of day, think of an operator, who, in making a ligature of a large artery, such as the humeral, placed his chance of discriminating the attendant nerve from the bloodvessel which he wished to tie, by appealing, not to his own anatomical knowledge, but to the feelings of his patient, as he touched the suspected structures. "Does it hurt you, yes or no." Would our surgical brethren not denounce and decry the capabilities of any man who, in operating, should require to have recourse to such imperfect and incompetent diagnosis? Would it be right and moral in a surgeon to deny to his patients the advantages of anæsthesia, in order that their sensations and sufferings should make up for his want of anatomical and operative knowledge.

But in saying this, do not, I pray you, for one moment suppose that I fancy that the argument which you adduce, betrays any want whatever, of the highest degree of operative skill on your part. Nothing could be further from my thoughts. And, to confess the truth, I do sincerely believe, that you yourself, while using the forceps, do not require to have recourse to any such rule as you here propound; and that in fact, the rule itself, and the objection to anæsthesia in midwifery which it contains, is an *after thought* on your part, which has only sprung up since the practice of anæsthesia was proposed. For, in looking over the excellent precepts which you have given, relative to the use of the forceps in the valuable work on midwifery, which you published a few years ago, (viz., the Philadelphia Practice of Midwifery), I find no trace or mention whatever of such a rule as you have quoted above in your letter to me. If that rule really formed, as you now state, the "safest and most trustworthy" guide in the operation, you would certainly have at least noticed it, or alluded to it in some way. In the precepts which you laid down in your work, you would assuredly not have forgotten that one rule, which, you say, is worth a " thousand other dogmas and precepts." And I think it would have been only the more incumbent upon you to have mentioned it, seeing that all other authors omit the notice of it.

I feel assured that when you come to reconsider " dispassionately " your opinions regarding the non-employment of anæsthesia in operative midwifery, you will alter those opinions. And when you come to employ anæsthesia in actual practice, in cases in which the forceps are used, you will find that instead of impeding the application of the instruments, the anæsthetic state very greatly facilitates it. It enables you to guide the forceps far more safely to their destination, because it enables you, without any pain to the patient, to introduce your fingers for this purpose, far more deeply between the head and maternal structures than you could do if the patient were awake and in her usual sensitive state. You, yourself, state, in your published work on midwifery, that care should be " taken to direct the point (of the forceps) by the two fingers as far as they can reach." (p. 300.) " If (you again observe,) any difficulty occurs in getting the second blade forwards enough, the two left fingers that are guiding it will serve to guide it edgeways into the proper position." Now, the state of anæsthesia, I repeat, gives you (as I have several times found,) the power of fulfilling these and other important rules, to an extent that never can be attained without it, and I am sure you will find them worth any " thousand dogmas, and precepts," derivable from the mere sensations of the patient.

Besides, the sensations, or rather the expression of them, would betray you constantly if you *did* place any dependance upon them. Under the same amount of pain, scarcely any two women would give you exactly the same expression of suffering. What one woman would loudly complain of, another would declare to be nought.

Before interfering instrumentally with the forceps, the labour has generally been allowed to endure for twenty or thirty long hours. After a poor patient has undergone such a protracted ordeal of pain and suffering, her mind is not, I fear, in general, in a very fit state to guide the operator by her sensations or directions.

At page 302 of your published work on midwifery, you state that "when the forceps are used, the patient's mind is naturally wound up to a state of great anxiety; it is strained (you observe) to the highest tension by the mere thought that she is under the operation." Now, putting entirely out of view, for the moment, the propriety of our saving our patients the increased corporeal agony attendant upon instrumental delivery, is it not, let me ask, our right and our duty as medical men, to save her, as we can do, from this trying state of mental anxiety at the time of operating? In most cases, she will have been suffering, and struggling on for many hours previously. Why, then, thus needlessly and greatly intensify both her mental anxiety and physical sufferings at the time of our own instrumental interference, when her strength alike of mind and body are perhaps little calculated to bear any increase of suffering, and above all, when the resources of our art furnish us with simple and certain means of saving her from the unnecessary endurance of the one state and of the other.

But, in instrumental delivery, besides greatly facilitating the application of the forceps, and relieving the patient from enduring the pains of the operation, and that "highest tension" of mind which is present during it, the state of anæsthesia saves her, I believe, also in a great measure from the effects of the shock of the operation, and thus gives her a better chance of recovery. If we omit it, we omit, I believe, not only a means of saving her from the sufferings attendant upon the operation, but a means of saving her from some of the dangers attendant upon it. When first publishing on the subject of anæthesia in midwifery, in February, 1847, I offered one or two observations on this point, which subsequent surgical statistics have amply fulfilled. In allusion to some cases of operative delivery, which I recorded, I observed: "The cases I have detailed, sufficiently show its value and safety in cases of operative midwifery. And here, as in surgery, its utility is certainly not confined to the suspension and abrogation of conscious pain, great as, by itself, such a boon would doubtlessly be. But, in modifying and obliterating the state of conscious pain, the nervous shock otherwise liable to be produced by such pain,—particularly whenever it is extreme, and intensely waited for and endured,—is saved to the constitution, and thus an escape gained from any evil consequences that are apt to follow in its train."

The observations which I have hitherto made, refer entirely to your opinion of anæsthesia in instrumental delivery. But,

2. *You object to anæsthesia in natural labours, because you hold that the pain of natural labour should not be annulled, and that it is calculated to promote the safety of the operation.*

You regard, you say, " the pain of a natural labour as a state, not by all possible means and always to be eschewed and obviated;" " a labour pain being (you declare,) a most desirable, salutary and conservative manifestation of life-force."

In the above expressions you make no distinction between the two separate and distinct elements of which a so-called labour-pain consists, viz.: 1, the contractions of the uterus, and 2, the sensations of pain resulting from these contractions. If you apply the language I have quoted to the first of these elements, the uterine contractions, (and which contractions are not annulled by anæsthetics,) I decidedly and entirely agree with you. If you apply it, however, to the sensations of pain produced by the uterine contractions, (and which sensations are annulled by anæsthetics,) I most decidedly and entirely dissent from your opinion.

In your work on midwifery, you make, correctly, the important distinction to which I refer. You state (p. 148,) that " the word (labour) is highly expressive of the violent and painful struggles and efforts of the woman." You add that " the essential element of labour is the contraction of the muscular fibres of the womb." And at page 303, in speaking of the strength of these uterine contractions, you observe, " let it be well borne in mind, that the expulsive powers of the womb are enormously great." In more than one place in your work, you allude to the intensity of the sensations of pain, (the pangs and agonies of travail, as you term them, p. 155); and at page 153 you speak of the " painful sensations " of the mother in the last part of labour, as so great in degree " as to be absolutely indescribable, and comparable to no other pain." In your still later work on Female Diseases, speaking of these pains—the pains of parturition—you observe, " Men cannot suffer the same pains as women. What (you continue,) do you call the pains of parturition ? There is no name for them but agony."

The muscular contractions of the uterus form, you say, the "essential element" of labour. In that opinion you and I are at one; and further, I quite agree, that this cannot be safely "eschewed and obviated" in natural labour, nor are they "eschewed and obviated" under the proper use of chloroform.

But the pain, the second element, is a non-essential element in the process. It is non-essential, because, 1st, labour, that is, the uterine contractions, are occasionally, though very rarely in the course of practice, seen to accomplish the full expulsion of the child with little or no pain. 2d. In whole tribes of the human race, as in some of the black tribes, little or no pain seems to be endured, if we may believe various authorities; and 3d, hundreds of women have, during the last year, been delivered with perfect safety, but without any pain, while placed under the influence of anæsthetic agents.

I hold the pain to be *non-essential*, and I protest against the truth of your opinion that "the pain of a natural labour is a state not by all possible means to be eschewed and obviated." On the contrary, I maintain that we omit and forego a weighty part of our professional duties whenever we forget the axiom of Bacon, that "it is the office of a physician, not only to restore health, but to mitigate pain and dolours." And as medical men we are called upon to mitigate and remove those "pangs and agonies of travail," (as you term them,) which, in degree, are in your own language, "absolutely indescribable, and comparable to no other pain,"—pain, for which there is "no other name but agony."

In your practice, you, like other medical men, constantly use measures to mitigate and relieve the pains of headache, of colic, of sciatica, of pleurodyne, of gout, rheumatism, and all the other innumerable "dolours" that flesh is heir to. Like other physicians, you deem it, I doubt not, your duty to wield the powers of your art, in order to free those that submit themselves to your medical care from these and other similar sufferings. But, if it is right for you to relieve and remove these pains, why is it not right for you also to relieve and remove the pains accompanying the act of parturition? I cannot see on what principle of philosophy, or morality, or humanity, a physician should consider it his duty to alleviate and abolish, when possible, the many minor pains to which his patients are subject; and yet should consider it improper to alleviate and abolish, when possible, pains of so aggravated a character that, in your own language, they are "absolutely indescribable, and comparable to no other pains,"—pains for which there is "no other name but agony."

3. *You object to anæsthesia in natural labour because you deem the pain of natural labour "a physiological pain."*

"The sensation of pain in labour is (you observe) a physiological relation of the power or force," and "to be in natural labour is the culminating point of the female somatic forces."

Now, for the reasons that I have already stated, I entirely doubt if we should look upon the severe sensations of pain endured by our patients as truly "physiological," for, as I have just stated, they are not essential to the mechanism and completion of the process in the white races of mankind, and they are absent to a great degree in the black. The severity of them could, I think, be easily proved to be the result of civilization, and, as I believe, of that increased size of the infantile head which results from civilization. Parturition is always physiological in its object, but not in some of the phenomena and peculiarities which attend upon it in civilized life.

But waiving this point or the discussion of it, let me state that even if I allowed all the intense pain of parturition to be "physiological pains," I cannot conceive that to be any adequate reason for our not relieving women from the endurance of them. Because nature has fashioned any particular physiological function in any particular manner, that, I opine, is no reason why the science and art of civilized life should not, when possible, alter and amend its workings. If it were improper for us, for instance, to intermeddle with the functions of the hair of the head, or of the skin generally, then all hats and other coverings for the scalp, all clothing and coverings for the body, should be at once abandoned and unconditionally condemned. If it were improper for us to alter and amend the functions of the eye, then all optical glasses, the telescope, the microscope, &c., must be thrown aside. And indeed not later than the 17th century, it was held and argued so in England. For in his history of the first beginning of the Royal Society of London, Sprat tells us that it was generally believed that this new experimental philosophy (namely the philosophical papers laid before the Society,) was subversive of the Christian faith, and many, he adds, mortally hated the newly invented glasses, the telescope and the microscope, as *atheistical inventions*, which prevented our organs of sight, and made everything appear in a new and false light. (D'Israeli.)

You argue as if supposing that we should not use means to eschew the pain of parturition, because that pain is physiological. When Columbus first discovered your mighty American continent, a large portion of the inhabitants were unprovided with any kind of dress or covering. "To most of them," says Robertson, "nature had not even suggested any idea of impropriety in being altogether uncovered." And I do think that men living in such a state could, against the fashion of dressing, use with far greater propriety and consistency than you or I, your own argument against anæsthetics in labour. Chloroform and ether should not be used in labour, (you argue,) because the pain against which they protect us is natural and physiological. No kinds of clothing or dress should be used, (the original Americans might have equally argued,) because the cold and heat against which they protect us are natural and physiological.

I have a letter lying before me on the subject of anæsthetics in midwifery, by a very highly and very justly esteemed teacher of midwifery in Dublin. "I do not (he writes) believe that any one in Dublin has as yet used ether in midwifery, the feeling is very strong against its use in ordinary cases, and merely to avert the ordinary amount of pain which the Almighty has seen fit,—and most wisely we cannot doubt—to allot to natural labour, and in this feeling I heartily and entirely concur."

The argument thus used, and so very well expressed by my Irish correspondent, is one which has often been adduced and repeated during the course of the past year. Some minds at first gave immense weight and importance to it. For my own part, I must confess that I never could view it as containing any great force. Look at it as applied to any other practice which happens to be sufficiently old and established: and then we will see it in its true import. Supposing, for example, it referred to the *first* introduction of carriages into use: It would then read thus,—I do not believe that any one in Dublin has as yet used a carriage in locomotion; the feeling is very strong against its use in ordinary progression, and merely to avert the ordinary amount of fatigue which the Almighty has seen fit—and most wisely we cannot doubt—to allot to natural walking, and in this feeling I heartily and entirely concur.

Nay, this frequently repeated argument against new innovations becomes not only, I think, ridiculous, but really almost irreverent when we look far backwards into the march of civilization, and apply it to any practices that are so very long established as to be very antiquated, and with which, therefore, the human mind has been long and intimately familiarized. Some one, but who I cannot pretend to say, no doubt first introduced the practice of wearing hats or bonnets or some covering for the head. Suppose this practice, however, strongly resisted as doubtlessly it was at first, then the argument of my Dublin friend against this innovation would read somewhat as follows:—I do not believe that any one in Dublin has as yet used a hat to protect his head; the feeling here is very strong against its use in ordinary weather, and merely to avert the ordinary amount of wetting and cold which the Almighty has seen fit—and most wisely we cannot doubt—to allot to mankind, and in this feeling I heartily and entirely concur.

Some day a canal will in all probability be made through the isthmus of Panama. It has, you are well aware, long been proposed to cut one; and there and thus unite the Atlantic and Pacific Oceans. When it was proposed in the 16th century, a priest of the name of Acosta brought forward the following reason against it. "I am (said he in writing in 1588) of opinion that human power should not be allowed to cut through the strong and impenetrable bounds which God has put between the two oceans, of mountains, and iron rocks, which can stand the fury of the raging seas. And if it were possible, it would appear to me very just that we should fear the vengeance of Heaven, for attempting to improve that which the Creator in his almighty will and providence, has ordained since the creation of the world." The arguments which are here brought forward by the earnest Spanish priest, against man meddling with and altering the impediments to navigation caused by the natural mechanism of the Isthmus of Panama, are essentially the same as those lately brought forward against man meddling with and altering the agonies caused by the natural mechanism of parturition in the civilized woman. We can all, perhaps, at this time of day, see through and smile at the character of the old priest's argument, with regard to the supposed impropriety of changing and cancelling, if possible, the natural obstruction produced by any isthmus. Some years after this, perhaps our descendants will equally see through and smile at the analogous modern argument in regard to the supposed impropriety of changing and cancelling, when possible, the physical suffering produced by a physiological function.

The truth is, all the tendencies of man in a civilized state of society are to intermeddle with and change, and, as he conceives, improve the action of almost every function in the body. And each such improvement has at the time of its introduction, been like the practice of anæsthesia, very duly denounced as improper, impious, &c., &c. I might refer to numerous such cases. Let me cite only one example. The human fingers are admirably constructed by our Creator for the function of seizing and lifting objects. The late Sir Charles Bell wrote a whole octavo volume—a Bridgewater Treatise—on the mechanism of the human hand as beautifully adapted for this and other functions. In the reign of the earlier Stuarts forks were introduced from the continent to assist our hands in the act or function of seizing and lifting the divided portions of meat, &c., that we wished to eat. But this was a very sad and uncalled for innovation upon the old and established physiological functions of the human fingers; and at the time it was as loudly opposed and decried, as the modern employment of anæsthetics in aiding the physiological function of human parturition. D'Israeli tells us that the use of forks was so much reprobated in some quarters, that some uncleanly preachers denounced it "as an insult on Providence not to touch our meat with our fingers. Nature herself has provided us with fingers of flesh and bone and nerve, and, consequently, is it not unnatural and impious in man to attempt, in his human pride and arrogance, to substitute for these, artificial metallic fingers of silver and steel!"

I repeat, all our tendencies and workings in the present state of civilization are attempts to intermeddle with and change and improve the action of almost every function in the economy. And assuredly if we use means in regard to the function of parturition, with the view of ameliorating the unnecessary, but, as you call them, " absolutely indescribable " pains that attend upon it, we would be doing nothing more than what you and I and all of us are ever doing, in most of the other natural or physiological functions of our own bodies.

Let me illustrate this last remark by one more example, for, as I have already said, it is only in this way that we can properly judge of the soundness or unsoundness of our views of novel points in theory or practice. You are well aware that the act of parturition has been often familiarly compared, as the late Professor Hamilton expressed it, " to the toils of a journey," and like it divided into stages. " The sufferings of the mother (says he) have been in most languages compared to those of travellers." Now, let us for a moment consider this natural simile between the function of parturition and the function of progression. You maintain that " labour is the culminating point of the female somatic forces." One of the most illustrious Presidents of your great American republic—Thomas Jefferson—makes in his memoir a remark of precisely the same import, regarding walking or progression. He describes the act of walking, but not exactly in the same words, as the kind of " culminating point of the human somatic forces."*

Few or none perhaps will question the abstract truth of Jefferson's observations on this point. But, because walking or progression is a " physiological " function, and the practice of it is reputed salutary, would this be with you a proper and sufficient reason for never setting aside or superseding in any way this " physiological ", state, in the same way as you insist, on the same grounds, that the physiological pain of labour should not be set aside or superseded ? Because progression is a natural condition, would this be any adequate reason for your medical advisers adopting your own arguments against anæsthesia in mid-

* Since writing the above I have turned up Jefferson's memoirs to get his own words. " Walking (says the American President) is the best possible exercise ; habituate yourselves to walk very far. The Europeans (he continues) value themselves on having subdued the horse to the uses of man ; but I doubt whether we have not lost more than we have gained by the use of this animal. No one has occasioned so much (as the horse) the degeneracy of the human body. Our Indians go on foot nearly as far in a day for a long day, as an enfeebled white does on his horse : and he (the Indian) will tire the best horses." (Memoirs, vol. i. p. 287.)

wifery, and insisting upon this, that, the next time you travelled from your own city of Philadelphia to the cities of Baltimore or New York, you should walk the distance on foot instead of travelling it by railway or other conveyance? What opinion would you form of the judgment of any medical adviser to whom you entrusted your own health, if, on going next time to the New York or Baltimore railway station, he should gravely and solemnly repeat to you as his patient what you tell your midwifery patients, and, in your own language, advise you to try to accomplish the intended journey on foot, as (to quote your own words,) "a desirable, salutary, and conservative manifestation of life-force?" And yet this would really be nothing more than making your *argumentum ad fœminam* an *argumentum ad hominem*.

You state, in a passage which I have already quoted, that even the agony accompanying instrumental delivery by the forceps, is a "physiological pain." I do not, I confess, see why the sufferings attendant on the use of the forceps, when the head is impeded by any cause of obstruction, should be regarded as a physiological pain, any more than the suffering attending the use of the catheter in obstruction from the prostate gland, or other morbid conditions of the urethra should be regarded as a "physiological pain." They are both operations intended to remove the natural contents of the respective viscera, when their operative removal becomes necessary.

But let us waive this point, and return again to the analogy between the functions of progression and parturition. Suppose that you plead with your medical advisers that, instead of insisting on your going on foot, they should allow you for once to take advantage of artificial assistance, and proceed on your journey from Philadelphia to Baltimore, or New York, by railway, because you were unable to walk the distance, in consequence of being incapacitated by a rheumatic knee, or a sprained ankle, or an inflamed or blistered toe, and they replied to you that you should not care for this, but still proceed and suffer, because the pain you might thus suffer, was (to use again your own language,) still only a "physiological pain." Would that argument be any adequate philosophic consolation under the endurance of your suffering? Or would you not laugh at the logic of your medical adviser, and take your seat in the railway in spite of his doctrine? And I have a fancy, that betimes, in midwifery, patients *will* have to adopt exactly the same line of practice under the analogous circumstances, and think, and act, too, in the same way.

[The remaining "objections" are unavoidably postponed until the next number.—Eds.]

